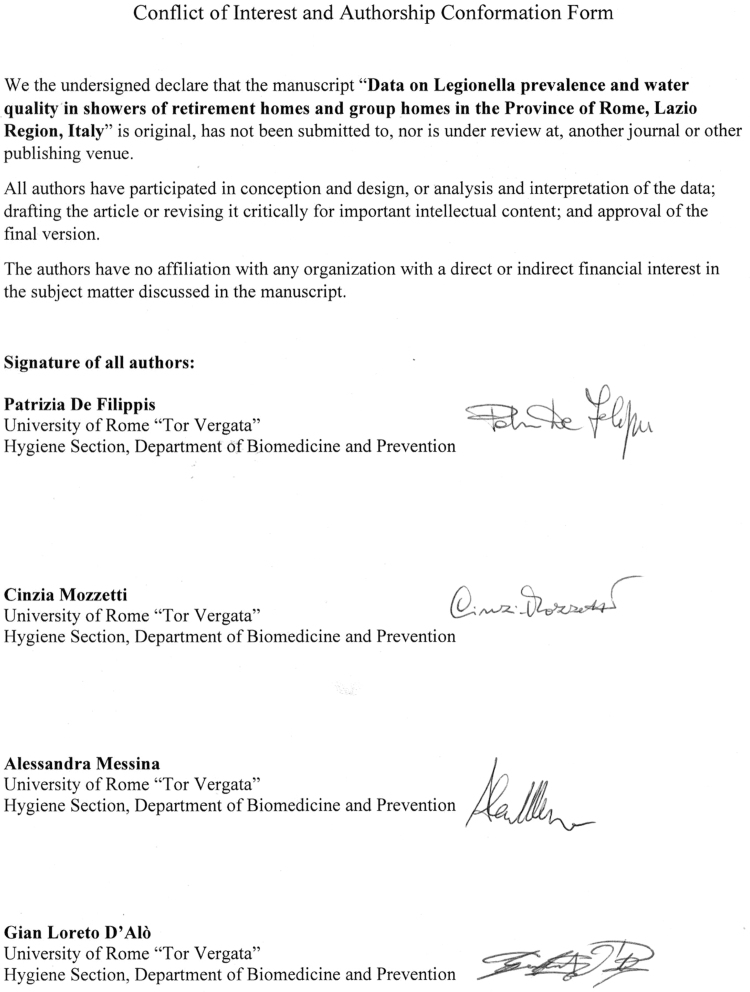# Data on Legionella prevalence and water quality in showers of retirement homes and group homes in the Province of Rome, Lazio Region, Italy

**DOI:** 10.1016/j.dib.2018.07.026

**Published:** 2018-07-17

**Authors:** Patrizia De Filippis, Cinzia Mozzetti, Alessandra Messina, Gian Loreto D’Alò

**Affiliations:** Section of Hygiene, Department of Biomedicine and Prevention, University of Rome “Tor Vergata”, Via Montpellier 1, 00133 Rome, Italy

**Keywords:** CFU, Colony Forming Units, HPC, Heterotrophic Plate Counts

## Abstract

The data presented in this article are related to the research paper titled "Prevalence of Legionella in retirement homes and group homes water distribution systems" (De Filippis, 2018) [3]. Most of the cases of Legionella infection are sporadic and occur in community-dwellers. Hot water and biofilm samples from the showerheads of 26 retirement homes and 9 group homes hosting closed communities were collected, in order to evaluate the prevalence of Legionella and generic water quality parameters (Heterotrophic Plate Counts at 22 °C and 37 °C). Samples were tested by culture method for the presence of Legionella. Confirmation and identification were carried out through Latex test and PCR. Further data about buildings’ number of floors and rooms were collected, and put in relation to the presence of Legionella through constructing contingency tables and performing exact fisher׳s or Chi-square tests. Cold (< 30 °C) water samples are analyzed apart.

**Specifications Table**

**Table:** 

Subject area	*Microbiology, Social assistance facilities*
More specific subject area	*Legionella, water quality, retirement homes, group homes*
Type of data	*Tables, graphs, figures, raw data*
How data was acquired	*Survey, Plate culture, Legionella Latex test (Oxoid ™) immunoassay, Polymerase Chain Reaction*
Data format	*Raw, analyzed*
Experimental factors	*Instantaneous water samples and biofilm samples to evaluate the exposure during the shower; sampling performed in facilities that host closed communities and at-risk individuals; high sensitivity and specificity Legionella identification.*
Experimental features	*Correlation between Legionella presence and/or load and generic water quality parameters (Heterotrophic Plate Counts at 22 °C and at 37 °C) were investigated. Relation between buildings’ characteristics and Legionella presence were investigated.*
Data source location	*Province of Rome, Lazio Region, Italy*
Data accessibility	*Data are included within this article*

**Value of the data**

• Data show the presence and the concentration of Legionella and the Heterotrophic Plate Count at 22 °C and 37 °C in samples gathered from showers of retirement homes and group homes.
• Data on Legionella presence and HPCs are shown together with information on the size of the analyzed structure (floors, rooms), water network system characteristics, the water sample temperature (< 30 °C; ≥ 30 °C), the type of population housed.
• Data provide information on the potential exposure of people living in retirement homes and group homes to Legionella contained in shower aerosols.
• Instantaneous sample method and biofilm sampling method used together help in simulating the theoretical user׳s exposure.

## 1 Data

This data article contains tables, figures and data files showing the results of the analysis of 140 water and biofilm samples collected from 26 retirement homes and 9 group homes.

*Excel spreadsheet (Raw data):* samples’ analyses results (HPCs, Legionella), together with all buildings’ and water systems’ available characteristics, are shown.

**Fig. 1:**
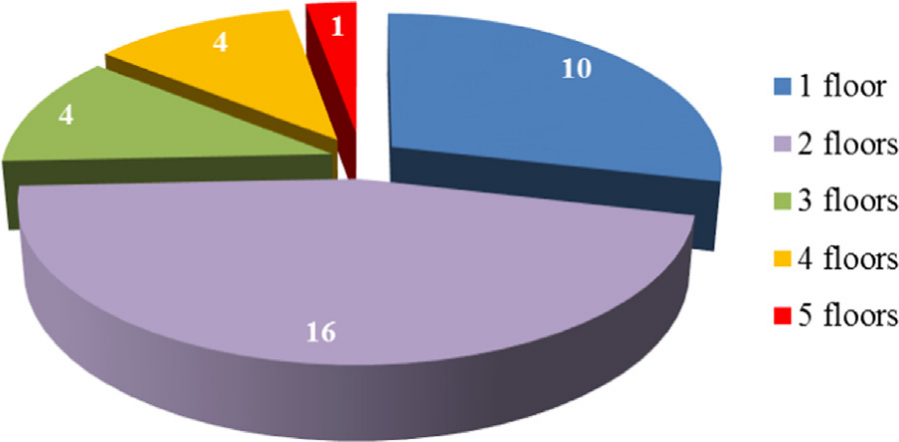
Distribution of the analyzed buildings based on the number of floors.

Fig. 1 shows how the analyzed buildings are distributed based on the number of floors, while in Table 1 this parameter is statistically put in relationship with the presence of Legionella.

**Fig. 2:**
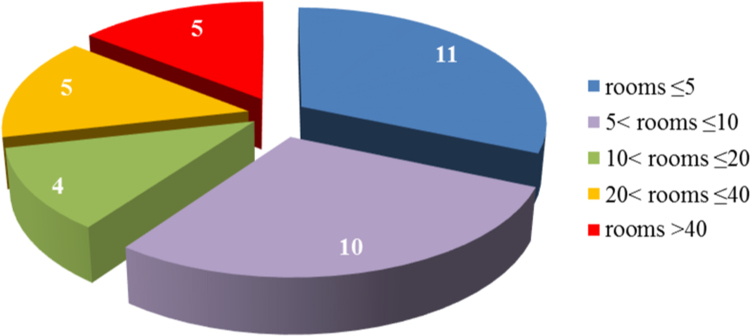
Distribution of the analyzed buildings based on the number of rooms.

Fig. 2 shows the distribution of the analyzed buildings based on the number of rooms, and these data are analyzed in Table 2 similarly to data on number of the floors.

In Table 3 the buildings are classified according to the number of rooms and floors, in relation to the presence of Legionella.

Table 4 shows the calculation of the number of buildings in which HPCs anomalies were found.

In Table 5 these findings are put in statistical relationship to the presence of Legionella.

Table 6 shows the characteristics of collected water samples (Temperature < 30 °C).

**Fig. 3:**
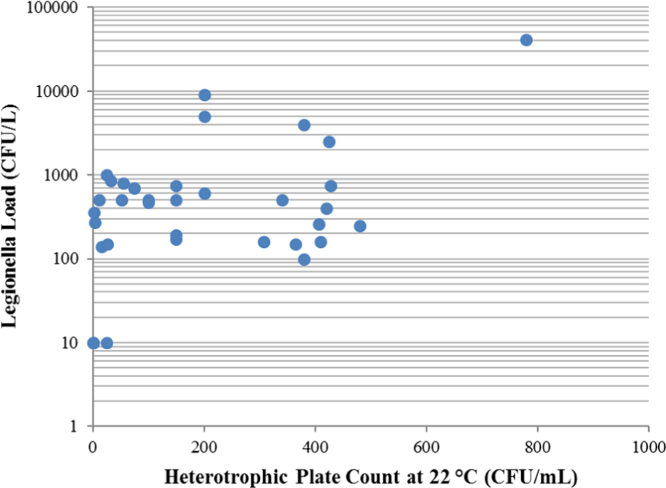
Scatter Plot showing the relationship between Legionella load (CFU/L) and the Heterotrophic Plate Count at 22 °C (CFU/mL). Pearsons’ *r* = 0.5458; *r*^2^ = 0.2979. 2 samples not included in the construction of the plot because the HPC 22 °C was over 800 CFU/mL, so that it couldn’t be determined with accuracy.

In Fig. 3 the Legionella loads (CFU/L) of the positive water samples are put in relationship with the HPCs at 22 °C (CFU/mL).

**Fig. 4:**
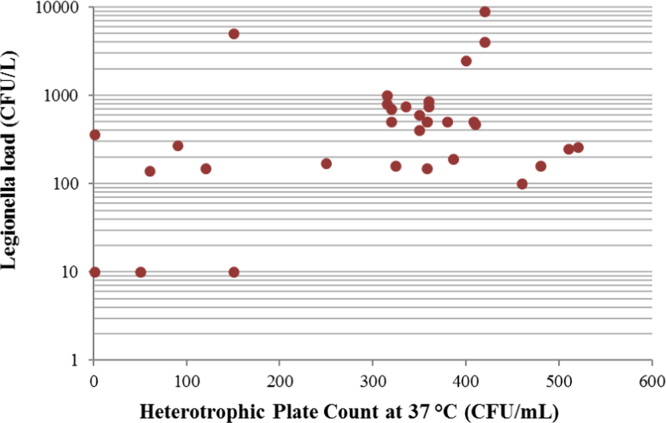
Scatter Plot showing the relationship between Legionella load (CFU/L) and the Heterotrophic Plate Count at 37 °C (CFU/mL). Pearsons’ *r* = 0.1489; *r*^2^ = 0.0222. 3 samples not included in the construction of the plot because the HPC 37 °C was over 800 CFU/mL, so that it couldn’t be determined with accuracy.

Similarly, in Fig. 4 Legionella load data are related to HPCs at 37 °C.

**Fig. 5:**
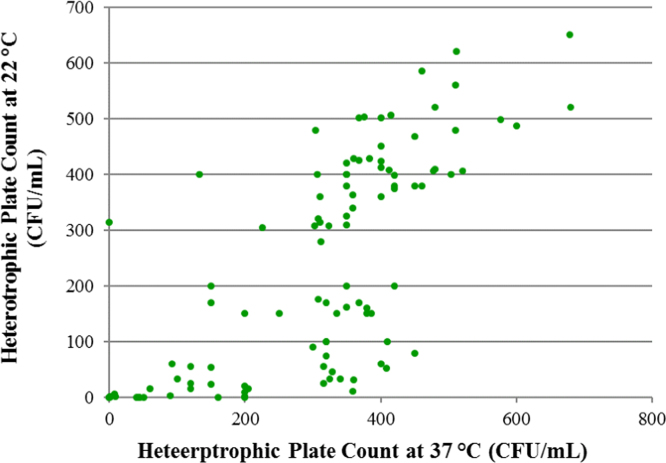
Scatter Plot showing the observed relationship between the Heterotrophic Plate Count at 37 °C and at 22 °C (CFU/mL). Pearsons’ *r* = 0.7293; *r*^2^ = 0.5319. Cold (< 30 °C) water samples included. 33 samples not included in the construction of the plot because the one of the HPCs was over 800 CFU/mL, so that it couldn’t be determined with accuracy.

Fig. 5 shows the relation between the HPC at 22 °C and at 37 °C for each sample.

Table 7 reports the estimation of the analyzed facilities’ number of beds, of hosted people and exposed population.

## 2 Experimental design, materials and methods

### 2.1 Samples collection

Water and biofilm samples were collected, from September 2015 to September 2016, from hot water distribution systems of 35 facilities (26 retirement homes and 9 group homes) in the area of Rome (Lazio, Italy). All the buildings were supplied from the public network. From every distal point (shower heads) we collected both a water and a biofilm sample.

### 2.2 Sampling method

All water samples without flaming the outlet port and without previously running the water namely “instantaneous sample”, to simulate the theoretical user׳s exposure [10].

All samples were collected by turning on the hot water, and the temperature was measured; however, some of them did not exceed 30 °C.

Legionella standard sampling procedures for water are reported in the [6] (Water quality–detection and enumeration of Legionella) and in the Italian Guidelines for the prevention and control of legionellosis [10], [6].

The samples were collected in 1 L sterile polyethylene bottles with 10% sodium thiosulphate to neutralize the chlorine (able to neutralize up to 5 mg/L of residual free and combined chlorine) [8,2].

Free chlorine concentrations in water samples were not measured in our study, and data on disinfection techniques used in the water systems were not available.

The bottles were transported to the laboratory for the microbiological analysis in suitable container, at room temperature and protected from direct light.

In addition to water samples, we collected biofilm samples from the surface and inside of all shower heads by the use of sterile swabs in alginate fiber (Swab Rinse Kit SRK, COPAN). Inner surfaces were sampled through the removal and disassembly of the showerheads. The swab is generally used to sample irregular surfaces and, due to the difficulty of calculating the sampled area, this type of investigation is considered a qualitative but not quantitative sampling method. The swab surface sampling technique is described in the [7] and the Italian Guidelines 2015 [10], [7]. The samples were stored for transport in an isotonic solution, that was part of the sampling kit, and that consisted of a buffered phosphate salts solution.

### 2.3 Microbiological analysis

#### 2.3.1 Water samples

Legionella isolation was performed in accordance with the ISO 11731 and the Italian Guidelines [10], [6], with minor modifications [4] to increase sensitivity in detecting Legionella. The water sample was filtered through 0.20 μm pore-sized cellulose nitrate filter (Sartorius). Filter was resuspended in 5 mL of the original water sample and shaken with vortex for 2 min to detach the bacteria. In order to reduce contamination by interfering microorganisms, the sample was held at 50 °C for 30 min. Then, an aliquot of 0.5 mL was spread on Legionella CYE agar base (Oxoid) with an addition of BCYE growth supplement and GVPC selective supplement (Oxoid). The inoculated plates were incubated at 37 ± 1 °C in 2.5% CO_2_ for 10 days and read every day. Suspected colonies were counted from each plate and subsequently confirmed by their inability to grow on CYE agar base without BCYE growth supplement. Finally, the colonies were evaluated with Legionella Latex Test Kit (Oxoid). This is a latex agglutination test for the identification of predominant Legionella species grown on plate media from patients with suspected Legionellosis or from environmental sources. The overall sensitivity of the test, as observed in the producer׳s trials, was 99%, while the overall specificity was 100% [5].

The test allows a separate identification of *L. pneumophila* serogroup 1, *L. pneumophila* serogroup 2–14 and detection of seven Legionella (polyvalent) species, which have been implicated in human disease: *L. longbeachae*, *L. bozemanii* 1 and 2, *L. dumoffii*, *L. gormanii*, *L. jordanis*, *L. micdadei*, and *L. anisa*. Each Kit contains positive and negative control to Legionella.

Each Kit contains positive and negative control to Legionella, which were performed each time the Kit was used. Furthermore, all positive colonies and a subset of negative colonies were confirmed by PCR (see Section 2.4).

The results were expressed in Colony Forming Units per liter (CFU/L), and the detection limit, based on the concentration factor and the volume of the inoculum, was 10 CFU/L. Accuracy of method is monthly checked through internal titered control.

An aliquot of each water sample was taken to determine the load of the Heterotrophic Plate Count (HPC) at 22 °C and 37 °C. These bacteria have been determined in duplicate by the pour plate method, using standard Plate Count Agar (Oxoid). The plates have been incubated at 37 °C for at least 48 h and at 22 °C for at least 72 h. The results have been expressed in CFU/mL [2,12].

In the Presidential Decree of 2001 (31/2001) [2] there are no threshold values for both HPCs at 22 °C and 37 °C to assess water potability. However, it is mandatory, as indicator parameter of the effectiveness of disinfection treatments, the research of HPT at 22 °C, which must not undergo "anomalous variations".

In the present work, anomaly in HPCs was defined as a large difference found in the HPCs at the different sampling points in the same building. Since we calculated also the HPC at 37 °C, the difference was considered to be “large” if were simultaneously detected at least one sample with an HPC ≤ 100 CFU/ml and another sample with HPC > 500 CFU/ml grown at the same temperature (22 °C or 37 °C).

#### 2.3.2 Biofilm samples

The swabs used for sampling biofilms were processed immediately. Every swab collected was held at 50 °C for 30 min to reduce contamination by interfering microorganisms. Then, an aliquot of 0.5 mL was spread on Legionella CYE agar base (Oxoid) with an addition of BCYE growth supplement and GVPC selective supplement (Oxoid). Legionella isolation and identification were performed following the same procedures for water samples.

### 2.4 PCR testing

Isolated positive Legionella colonies, both from water and biofilm samples, have beed also confirmed by Polymerase Chain Reaction (PCR) assay, according to the protocol of Van der Zee et al. [13]. The primer set used, LEG1 (50TACCTACCCTTGACATACAGTG-30) and LEG2 (50-CTTCCTCCGGTTTGTCAC-30), was derived from the 16S rRNA gene sequence and used to amplify a 200 bp DNA fragment specific for all Legionella species. The PCR reaction mixture, 25 μL final volume, contained 10 pmol of each primer, LEG1 and LEG 2, 200 μM of each dNTP, 3 mM MgCl2, and 2 U AmpliTaq Gold polymerase in 1 × PCR buffer (Promega). Samples were preheated for 10 min at 95 WC, followed by 40 cycles of 30 s at 94 WC, 30 s at 60 WC, and 30 s at 72 WC, with a final extension of 5 min at 72 WC. A negative and positive control was included in each PCR run. Amplified DNA was detected by agarose gel electrophoresis and ethidium bromide staining.

The test was performed on all colonies that tested positive at the Legionella Latex Test Kit (Oxoid) and on some colonies showing morphological characteristics similar to those of Legionella, growing only on selective medium, but negative on the agglutination test. However, all colonies in this subgroup were also negative for PCR.

### 2.5 Exposed population estimation

Exposed population data was inferred through Italian National Institute of Statistics data [9] on ratio between the number of people hosted and the number of beds available in social-assistance and socio-health facilities. The number of facilities’ rooms was multiplied by a factor of 1.(3), based on our available data about the composition of rooms (approximately 1 room with 2 beds for every 3 rooms) to calculate the number of beds in each building. Then, to calculate the number of people hosted in the facilities, the number obtained was multiplied by a factor of 0.95. This last factor was obtained from the ratio between the number of available beds and the number of people hosted in social-assistance and social-healthcare facilities in central Italy [9]. Finally, the number of exposed people was obtained multiplying the number of people hosted in the facility by the percentage of samples that tested positive for Legionella in the same facility. We have chosen this method of estimation, deliberately conservative, not being able to infer with certainty from our data the presence of Legionella in the whole water system, since we collected our water and biofilm samples with the only method of pre-flushing (instantaneous sampling).

### 2.6 Statistical analysis

All statistical tests were 2-sided, with statistical significance set at 0.05. Continuous variables were summarized using descriptive statistics and expressed as average and standard deviation (SD), as appropriate; comparison between two means have been made by using Student׳s *t*-test. Categorical data are expressed in percentages or summarized in contingency tables. When possible, variables were categorized into dichotomous ones. Odds ratios (OR) and 95% confidence intervals (CI) were calculated to assess categorical risk variables associated with microbial contamination. The qualitative analysis of categorical data has been performed through the construction of contingency tables, and the application of Fisher׳s exact test or Chi-square test, when appropriate.

In order to analyze the correlation of two quantitative variables, the values of the variables were used to build a scatter plot, and the Pearson product-moment correlation coefficient was computed.

Statistical analysis was performed using STATA version 13 [11].

Water samples whose temperature was < 30 °C were statistically analyzed apart. In fact, in our previous work [4] Legionella has been found only in 2 out of the 49 samples (4.0%) characterized by a temperature < 30 °C, and an OR of 12.7 (95% CI: 2.9–55.2) between hot and cold water samples for Legionella positivity was observed. However, in other works a higher positivity was found among samples with a temperature ≤ 20 °C too [1]. For this reason, in this work we aimed to confirm or rebut our previous findings about the infrequent presence of Legionella in this group of samples.

## 3 Results

See Fig. 1, Fig. 2, Fig. 3, Fig. 4, Fig. 5 and Table 1, Table 2, Table 3, Table 4, Table 5, Table 6, Table 7. Additional results of the present study are shown in the main paper (De Filippis [3]).

**Table 1:** Analyzed building׳s number of floors related to the presence of Legionella.

**Building׳s floors, *n***	**Legionella presence (*n* expected) [cell *χ*2]**	**Legionella absence (*n* expected) [cell *χ*2]**	***Row Totals***
**1**	**2** (2.57) [0.13]	**8** (7.43) [0.04]	10
**2**	**3** (4.11) [0.30]	**13** (11.89) [0.10]	16
**3**	**1** (1.03) [0.00]	**3** (2.97) [0.00]	4
**4 or 5**	**3** (1.29) [2.29]	**2** (3.71) [0.79]	5
*Column totals*	9	26	35

The chi-square statistic is 3.6552. The *p*-value is 0.3012.

**Table 2:** Analyzed building׳s number of rooms related to the presence of Legionella.

**Building׳s rooms, *n***	**Legionella presence (*n* expected) [cell *χ*2]**	**Legionella absence (*n* expected) [cell *χ*2]**	***Row totals***
***n* ≤ 5**	**2** (2.83) [0.24]	**9** (8.17) [0.08]	11
**5 < *n* ≤ 10**	**1** (2.57) [0.96]	**9** (7.43) [0.33]	10
**10 < *n* ≤ 20**	**1** (1.03) [0.00]	**3** (2.97) [0.00]	4
**20 < *n* ≤ 40**	**4** (1.29) [5.73]	**1** (3.71) [1.98]	5
**> 40**	**1** (1.29) [0.06]	**4** (3.71) [0.02]	5
*Column totals*	9	26	35

The chi-square statistic is 9.4197. The *p*-value is 0.051424.

**Table 3:** Percentage of buildings positive for the presence of Legionella based on the number of building׳s floors and rooms.

**Building׳s rooms, *n***	***Legionella*****presence, %**	**Building׳s floors, *n***	***Legionella*****presence, %**
*n* ≤ 5	18.2	1	20.0
5 < *n* ≤ 10	10.0	2	18.8
10 < *n* ≤ 20	25.0	3	25.0
20 < *n* ≤ 40	80.0	4	50.0
> 40	20.0	5	100.0

**Table 4:** HPCs anomalies: buildings in which a large difference^a^ was found in the Heterotrophic Plate Counts (HPCs) at the different sampling points.

	**HPC 37 °C**	**HPC 22 °C**	**At least one between HPC 22 °C and HPC 37 °C**
Buildings, *n* (%)^b^	7 (21)	10 (30)	11 (33)

a Defined as the simultaneous detection, in the same building, of at least one sample with an HPC ≤ 100 CFU/ml and another sample with HPC > 500 CFU/ml.

b Calculated on a total of 33 buildings, since in 2 buildings only 1 sample was gathered, so that the definition of large difference was not applicable.

**Table 5:** Buildings in which a large difference^a^ a was found in the HPCs at the different sampling points, related to the presence of Legionella.^b^

**Large differences**^a^**in HPCs found**	***Legionella*****positive buildings, *n***		***Legionella*****negative buildings, *n***	**Total**
Yes	**4**		**7**	11
No	**5**		**17**	22
Total	9		24	33
The two-tailed *P* value equals 0.4376^c^				

a Defined as the simultaneous detection, in the same building, of at least one sample with an HPC ≤ 100 CFU/ml and another sample with HPC > 500 CFU/ml.

b Calculated on a total of 33 buildings, since in 2 buildings only 1 sample was gathered, so that the definition of large difference was not applicable.

c Fisher׳s exact test was performed.

**Table 6:** Cold (< 30 °C)^a^ water samples main characteristics: microbiology and buildings of collection.

**Cold water samples, *n***	**Legionella positive, *n* (%)**	**HPC 37 °C, median (IQR), CFU/mL**	**HPC 22 °C, median (IQR), CFU/mL**	**Building floors׳ number, median (IQR)**	**Building rooms׳ number, median (IQR)**
16	0 (0%)	485 (310 - > 800)	573 (173 - > 800)	10 (6–12.5)	2 (2–2)

a In all the samples collected and here described the observed temperature was <15 °C.

**Table 7:** Estimated number of beds, of hosted people and of exposed population in the analyzed structures.

**Building ID**	**Building׳s type**	**Building׳s rooms**	**Estimated beds**^a^**, *n***	**Estimated hosted people**^b^**, *n***	**Legionella presence, % of samples (%)**	**Exposed people**^c^
**1**	Group home	50	67	63	0	0
**2**	Group home	50	67	63	0	0
**3**	Retirement home	3	4	4	0	0
**4**	Retirement home	3	4	4	0	0
**5**	Retirement home	10	13	13	0	0
**6**	Retirement home	10	13	13	75	10
**7**	Group home	8	11	10	0	0
**8**	Retirement home	12	16	15	0	0
**9**	Retirement home	40	53	51	50	26
**10**	Retirement home	62	82	78	70.6	55
**11**	Retirement home	20	27	25	77.8	19
**12**	Retirement home	3	4	4	0	0
**13**	Group home	8	11	10	0	0
**14**	Retirement home	28	37	35	75	26
**15**	Retirement home	10	13	13	0	0
**16**	Retirement home	10	13	13	0	0
**17**	Retirement home	10	13	13	0	0
**18**	Retirement home	5	7	6	0	0
**19**	Group home	3	4	4	0	0
**20**	Group home	3	4	4	0	0
**21**	Retirement home	5	7	6	100	6
**22**	Retirement home	5	7	6	0	0
**23**	Retirement home	15	20	19	0	0
**24**	Group home	5	7	6	75	5
**25**	Retirement home	42	56	53	0	0
**26**	Retirement home	5	7	6	0	0
**27**	Retirement home	40	53	51	75	38
**28**	Retirement home	10	13	13	0	0
**29**	Retirement home	5	7	6	0	0
**30**	Retirement home	10	13	13	0	0
**31**	Retirement home	38	51	48	50	24
**32**	Retirement home	40	53	51	0	0
**33**	Group home	20	27	25	0	0
**34**	Retirement home	60	80	76	0	0
**35**	Group home	6	8	8	0	0
**Total**	**/**	**654**	**870**	**826**	**/**	**209**

a Estimation based on available data on the beds/rooms ratio: we considered the ratio = 1.33.

b Estimation based on Italian National Institute of Statistics data [9] on ratio between the number of people hosted and the number of beds available in social-assistance and socio-health facilities; we considered a ratio = 0.95.

c Calculated multiplying the number of people hosted in facilities from which Legionella was isolated for the ratio of samples found positive for the presence of Legionella in the same facility.

## Figures and Tables

**Fig. S1:**